# Outbreak of postpartum group a Streptococcus infections on a labor and delivery unit

**DOI:** 10.1017/ice.2024.82

**Published:** 2024-09

**Authors:** Michael Haden, Christina Liscynesky, Nora Colburn, Justin Smyer, Kimberly Malcolm, Iahn Gonsenhauser, Kara M. Rood, Patrick Schneider, Michele Hardgrow, Preeti Pancholi, Keelie Thomas, Anita Cygnor, Oluseun Aluko, Elizabeth Koch, Naomi Tucker, Jade Mowery, Eric Brandt, Katie Cibulskas, Marika Mohr, Srinivas Nanduri, Sopio Chochua, Shandra R. Day

**Affiliations:** 1 Department of Internal Medicine, Division of Infectious Diseases, University of Colorado Anschutz Medical Campus, Denver, CO, USA; 2 Department of Internal Medicine, Division of Infectious Diseases, The Ohio State University Wexner Medical Center, Columbus, OH, USA; 3 Clinical Epidemiology, The Ohio State University Wexner Medical Center, Columbus, OH, USA; 4 Lee Memorial Health System, Fort Myers, FL, USA; 5 Department of Obstetrics and Gynecology, The Ohio State University Wexner Medical Center, Columbus, OH, USA; 6 Occupational Health and Wellness, The Ohio State University Wexner Medical Center, Columbus, OH, USA; 7 Department of Pathology, The Ohio State University Wexner Medical Center, Columbus, OH, USA; 8 Clinical Microbiology, The Ohio State University Wexner Medical Center, Columbus, OH, USA; 9 Department of Women and Infants, The Ohio State University Wexner Medical Center, Columbus, OH, USA; 10 Columbus Public Health, Columbus, OH, USA; 11 The Ohio Department of Health Laboratory, Reynoldsburg, OH, USA; 12 The Ohio Department of Health, Columbus, OH, USA; 13 Respiratory Diseases Branch, Division of Bacterial Diseases, Centers for Disease Control and Prevention, Atlanta, GA, USA

## Abstract

A healthcare-associated group A Streptococcus outbreak involving six patients, four healthcare workers, and one household contact occurred in the labor and delivery unit of an academic medical center. Isolates were highly related by whole genome sequencing. Infection prevention measures, healthcare worker screening, and chemoprophylaxis of those colonized halted further transmission.

## Introduction

*Streptococcus pyogenes*, also known as group A *Streptococcus* (GAS), causes both noninvasive and invasive infections including bacteremia, necrotizing fasciitis, and streptococcal toxic shock syndrome. Although invasive GAS infections account for a small proportion of total GAS disease, they can result in significant morbidity and mortality with a case fatality rate of 11.7% based on national epidemiologic data.^1,2^

Human skin and mucous membranes are the primary reservoir for GAS, with colonization of the throat being the most common; colonization of the skin, rectum, and vagina occurs to a lesser extent.^3–5^ While carriage rates are much higher in school-aged children, adult colonization is of particular concern, especially among healthcare workers (HCWs), given the risk for potential healthcare-associated spread of infection.^6^ Postpartum women have a 20-fold increased incidence of invasive GAS infection compared with nonpregnant women due to disruption of cutaneous or mucosal barriers during delivery, with approximately 220 postpartum cases occurring annually in the United States.^3,7^ GAS can be community-acquired from exposure or colonization with GAS prior to delivery or healthcare-acquired via transmission from a colonized HCW. Given the risk for healthcare-acquired infection and severity of disease for postpartum GAS infections, a prompt epidemiological investigation is recommended once a single case of postpartum GAS infection has been identified.

An epidemiological investigation was performed at a large academic medical center beginning in June 2019 to investigate six cases of invasive postpartum GAS infections occurring over four months.

## Methods

### Case definition

According to the 2002 Centers for Disease Control and Prevention (CDC) guidelines, postpartum invasive GAS is defined as isolation, during the postpartum period, of GAS in association with a clinical postpartum infection (e.g., endometritis) or from either a sterile site or wound infection. The postpartum period of interest includes all inpatient days and the first 7 days after discharge.^3^

### Investigational methodology

Case patients were identified by clinical epidemiology following notification of GAS from clinical cultures in the electronic medical record (EMR) prompting further investigation for postpartum patients. The EMR was utilized to compile a list of HCWs who had contact with case patients up to the date GAS infection was identified. HCWs with case-patient contact were evaluated for GAS colonization risk factors (recent illnesses, sick contacts, skin or soft tissue infections, or open draining wounds). Occupational health collected screening cultures from HCWs throat, vagina, peri-rectal area, and any open skin wounds; vaginal and peri-rectal cultures were self-collected. Household contacts from HCWs associated with multiple cases underwent this same screening process. We defined a carrier as an asymptomatic individual whose screening culture grew GAS. GAS isolates from case patients and HCW carriers or their close contacts were sent to the Streptococcus Laboratory at CDC for *emm* typing and whole genome sequencing (WGS) (Supplemental Material). The investigation was completed under the authority of the Quality Department and is exempt from Institutional Review Board (IRB) review.

## Results

**Figure 1.**
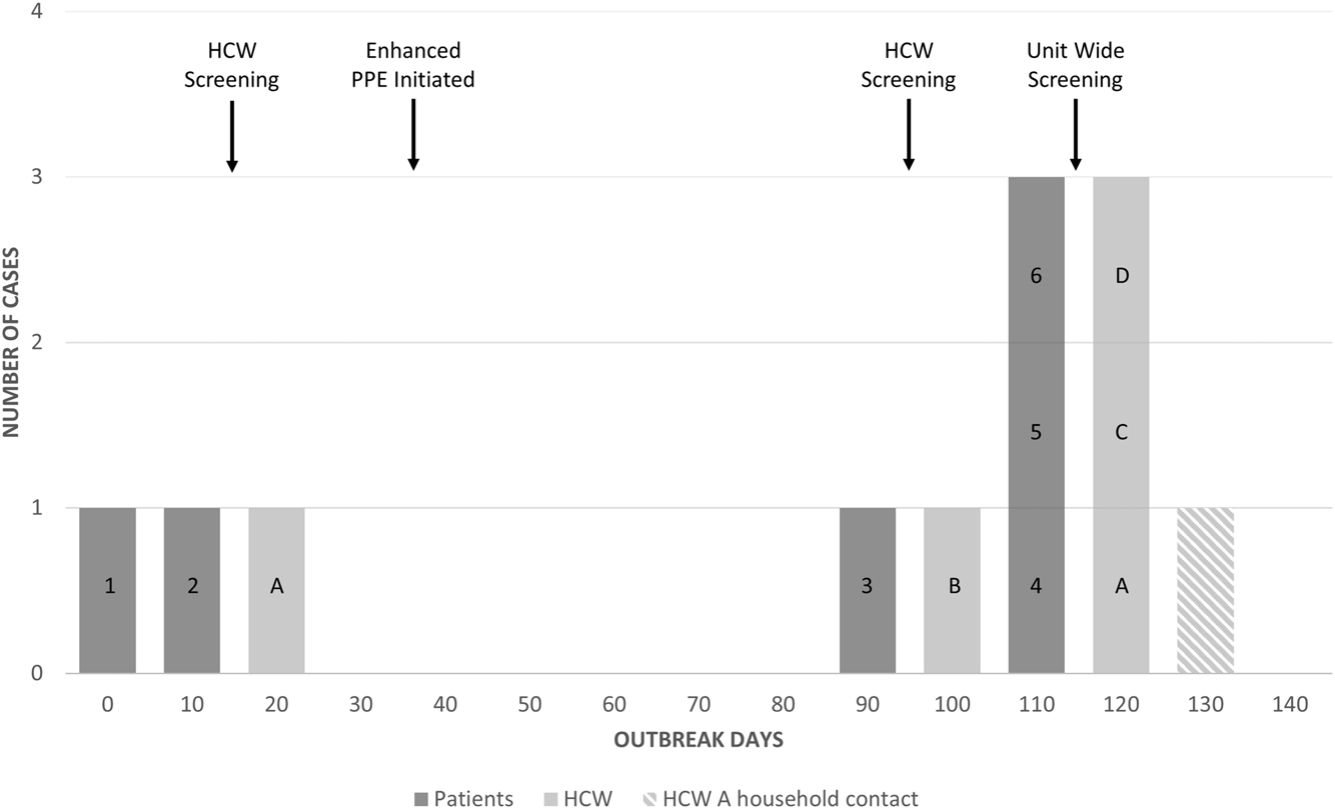
Outbreak epidemiologic curve and interventions.

During a period of 119 days, a total of 6 patients developed healthcare-acquired postpartum invasive GAS infection (Figure 1). The case-patients all had a spontaneous vaginal delivery within the preceding 7 days of symptoms onset. Six patients had endometritis with GAS from vaginal cultures, 4 patients also had GAS bacteremia and 1 patient developed toxic shock syndrome. All patients survived and were discharged in good condition.

A total of 43 HCWs were screened following patients 1 and 2, with a single HCW (HCW A) found to have GAS colonization (Figure 1) which was successfully eradicated with chemoprophylaxis. Following case 2, new requirements for personal protective equipment (PPE) were implemented including facemask, gown, and gloves for all HCWs present during delivery.

Patient 3 occurred 96 days into the outbreak. An additional 9 HCWs were screened following this case, with another HCW (HCW B) found to have GAS colonization (Figure 1), which was successfully eradicated with chemoprophylaxis.

The outbreak persisted with 3 additional patients (patients 4, 5, 6) identified 119 days into the outbreak. At this time mass screening of all HCWs involved in the labor and delivery unit was instituted. A total of 681 HCWs completed screening and 12 were found to be colonized with GAS. Three of the colonized HCWs (HCW A, C, D) were identified with the outbreak strain including HCW A, which prompted screening of their household contacts. One household contact of HCW A was found to be colonized with the outbreak strain (Figure 1). All received chemoprophylaxis and were successfully eradicated.

### Laboratory investigation

All clinical GAS isolates from the 6 invasive GAS case patients, 13 colonized HCWs, and 1 household contact were sent to the CDC Streptococcus Laboratory for WGS. The outbreak strain was identified as *emm*28 type, with all 6 isolates from case patients belonging to this type. Across the different rounds of screening, 4 HCWs (HCW A screened positive twice) and 1 HCW household contact were colonized with the outbreak GAS strain belonging to *emm*28 type. All outbreak-associated isolates clustered on a single branch of the phylogenetic tree and were highly related with a pairwise difference of 0–2 Single Nucleotide Polymorphisms (SNPs).

## Discussion

The outbreak described here involved 6 patients who developed healthcare-acquired invasive postpartum GAS infection acquired from 4 asymptomatic HCWs who were colonized with the outbreak strain. WGS of GAS isolates identified the outbreak strain as *emm*28 type, which is of particular significance as *emm*28 type GAS is a common cause of infection in pregnant and postpartum women and is significantly associated with puerperal sepsis.^7,8^ This is thought to be due to a mobile genetic element of apparent group B *Streptococcus* origin leading to increased tropism for vaginal tissue.^9^

Asymptomatic GAS colonization among adults is much less than that of children. In one study among military trainees the baseline colonization rate was 2.4%, though notably increased to 4.8% in part due to close quarters living conditions.^10^ During our unit-wide screening the overall colonization rate among staff was 1.8% (12/681).

Our investigation initially identified a single HCW (HCW A) with GAS colonization identified in oral, vaginal, and rectal cultures. Despite being heavily colonized, they were decolonized successfully per CDC guidelines including negative follow-up testing.^4^ The other HCWs colonized with the outbreak strain were positive in oral cultures only and all had negative testing following completion of chemoprophylaxis. Despite adhering to CDC guidelines for GAS investigation the outbreak continued, spanning 119 days. It is notable that all HCWs colonized with the outbreak strain worked the same shift; therefore, we speculate GAS spread persisted due to two factors: among HCWs in the workplace due to close contact in common areas and reacquisition of GAS colonization in HCW A following successful chemoprophylaxis due to a GAS positive household contact. Transmission to patients was only halted following mass screening of all HCWs and household contacts of HCW A, and chemoprophylaxis of those colonized, in addition to continued infection prevention measures, including PPE for all HCWs who entered patient rooms during delivery and regular audits for PPE and hand hygiene compliance. Mass screening was done over a short period of time, which allowed for identification and interruption of the ongoing transmission of the outbreak strain.

Our investigation highlights the importance of prompt identification of potential postpartum GAS outbreaks, along with strong lines of communication between patient care teams, hospital leadership, infection control, and the microbiology lab as well as coordination with local and state health departments.

## Supporting information

Haden et al. supplementary material Haden et al. supplementary material

## Supplementary material

The supplementary material for this article can be found at https://doi.org/10.1017/ice.2024.82.
