# Supplementary material for: Outbreak of postpartum group a Streptococcus infections on a labor and delivery unit
Source: Infect Control Hosp Epidemiol. 2024 May 14;45(9):1130–2. doi: 10.1017/ice.2024.82 (PMC11518660; doi:10.1017/ice.2024.82)
Supplement: Haden et al. supplementary material [file S0899823X24000825sup001.docx]

**Detailed Laboratory methods**

The *emm* type of each isolate was first determined using a sequential quadriplex real-time PCR-based method.^11^ Isolates with the outbreak *emm* type were then subject to whole genome sequencing (WGS) using methods reported previously.^12^ Briefly, GAS strains were cultured on Trypticase soy agar (TSA) supplemented with 5% sheep blood and incubated overnight at 37°C in 5% CO2. Genomic DNA for short read WGS was extracted on QIAcube HT using a modified QIAamp DNA QIAcube HT Kit protocol (Qiagen, Inc., Valencia, CA). Nucleic acid concentration was quantified by Invitrogen™ Qubit™ assay (Thermo Fisher Scientific Inc., USA) and samples were sheared using Covaris LE220 ultrasonicator (Covaris, Inc., Woburn, MA) programmed to generate 500bp fragments. Libraries were constructed on the SciCloneG3 (PerkinElmer Inc., Waltham, MA) using sparQ DNA Library Prep kit (Quantabio, Beverly, MA) with dual indexes (Illumina Inc., San Diego, CA) and quantified by KAPA qPCR library quantification method (Kapa Biosystems Inc., Wilmington, MA). WGS was performed by Illumina Next Generation Sequencing technology using MiSeq v3 600 cycle kit. Sequences were analyzed using the Streptococcus Laboratory bioinformatics pipeline and pairwise single nucleotide polymorphism (SNP) distances were generated for the core genome shared between isolates, employing kSNP3.0 with a kmer size of 19, and the MEGA7 program.^12,13,14^

11. Velusamy S, Jordak K, Kupor M, Chochua S, McGee L, Beall B. Sequential Quadriplex Real-Time PCR for Identifying 20 Common *emm* Types of Group A *Streptococcus*. Burnham CAD, ed. *J Clin Microbiol*. 2020;59(1):e01764-20. doi:10.1128/JCM.01764-20

12. Metcalf BJ, Chochua S, Gertz RE, et al. Using whole genome sequencing to identify resistance determinants and predict antimicrobial resistance phenotypes for year 2015 invasive pneumococcal disease isolates recovered in the United States. *Clin Microbiol Infect*. 2016;22(12):1002.e1-1002.e8. doi:10.1016/j.cmi.2016.08.001

13. Gardner SN, Slezak T, Hall BG. kSNP3.0: SNP detection and phylogenetic analysis of genomes without genome alignment or reference genome: Table 1. *Bioinformatics*. 2015;31(17):2877-2878. doi:10.1093/bioinformatics/btv271

14. Kumar S, Stecher G, Tamura K. MEGA7: Molecular Evolutionary Genetics Analysis Version 7.0 for Bigger Datasets. *Mol Biol Evol*. 2016;33(7):1870-1874. doi:10.1093/molbev/msw054
